# Cognitive performance and long-latency auditory evoked potentials: a study on aging 

**DOI:** 10.6061/clinics/2021/e1567

**Published:** 2021-01-18

**Authors:** Maria de Fátima Ferreira de Oliveira, Pedro de Lemos Menezes, Aline Tenório Lins Carnaúba, Liliane Desgualdo Pereira, Kelly Cristina Lira de Andrade, Ana Claudia Figueiredo Frizzo, Ilka do Amaral Soares

**Affiliations:** I Universidade Estadual de Ciencias da Saude de Alagoas (UNCISAL), Maceio, AL, BR; II Rede Nordeste de Biotecnologia, Universidade Federal de Alagoas (RENORBIO/UFAL), Maceio, AL, BR; III Centro Universitario (CESMAC), Maceio, AL, BR.; IV Faculdade de Medicina, Universidade Federal de Sao Paulo (UNIFESP), Sao Paulo, SP, BR; V Faculdade de Medicina de Ribeirao Preto, Universidade de Sao Paulo, Sao Paulo, SP, BR; VI Universidade Estadual Paulista Julio de Mesquita Filho, Marilia, SP, BR; VII Universidade Federal de Pernambuco (UFPE), Recife, PE, BR

**Keywords:** Evoked potentials, Cognitive Dysfunction, Aging.

## Abstract

**OBJECTIVE:**

To evaluate the relationship between cognitive performance and long-latency auditory evoked potentials in an elderly population.

**METHODS:**

The sample consisted of adults between 20 and 58 years of age and elderly adults between 60 and 70 years of age. The screening procedures adopted were an inspection of the external auditory canal, tonal and vocal audiometry, tympanometry, brain stem auditory evoked potential, the Montreal Cognitive Assessment test, and long-latency auditory evoked potential.

**RESULTS:**

The latency and amplitude values of cortical components by age group showed significant differences under the following conditions: (i) signals evoked by the speech stimulus /da/ and by the pure-tone stimulus at 2,000 Hz for the N2 amplitude (*p*=0.008 and *p*=0.001, respectively) , which were both higher for adults, and (ii) signals evoked by the speech stimulus /da/ for N1 latency (*p*=0.018) and by the pure-tone stimulus at 2,000 Hz for P2 latency (*p*=0.017), which were both higher in the elderly population. The cognitive component (P300) showed a significant difference when evoked by speech stimuli, with higher latency in the elderly population (*p*=0.013). When correlated with cognitive processes, the latency and amplitude of cortical potentials showed direct and medium-strength correlations between abnormal scores obtained on the Montreal Cognitive Assessment test and P2 amplitude (*p*<0.001 and r=0.452).

**CONCLUSION:**

There is a relationship between long-latency potentials and cognitive performance in the elderly, which was observed by the increase in the P2 amplitude and the impairment of the process of sound decoding.

## INTRODUCTION

In several countries, economic development has resulted in an increased life expectancy. According to the latest census, the life expectancy in Brazil increased for men and women, reaching 75.8 and 79.4 years, respectively. The most likely explanation for this is the improvement of infrastructure for health promotion, health care, and scientific and technological developments in different areas. However, new challenges emerge because more people are exposed to degenerative diseases associated with aging (1,2).

Age-related hearing loss (presbycusis) is a pathological process caused by genetic factors and affected by the preexisting conditions of an individual (systemic disease) and environmental factors (noise and ototoxic agents). Symptoms of presbycusis include decreased auditory sensitivity to high- and low-pitched sounds and difficulties in speech discrimination, especially in noisy environments. Changes in hearing thresholds are observed as a result of damage to peripheral auditory receptors and the cochlear nerve (3).

One of the most frequent complaints of elderly people is “being able to hear, but unable to understand the speech”. This symptom is common in patients with high-pitch hearing loss; however, it is also routinely reported in individuals with normal hearing thresholds or those with mild hearing loss. Changes in the nervous system resulting from aging have been recognized as an aggravating factor in the understanding of speech (4).

The hearing difficulties of elderly individuals include difficulties in sound localization, reduced speech recognition in noisy environments, difficulties in temporal processing and the perception of rapid changes in speech segments, and decreased attention. Studies have described difficulties in speech perception among elderly individuals compared to younger individuals (5).

Interest in the relationship between aging and auditory processing has increased in recent years, especially in the field of audiology because of the elderly individuals with normal peripheral auditory integrity or those who use hearing aids with adequate functional gain for hearing loss but present auditory manifestations incompatible with such hearing loss (6).

Auditory evoked potentials (AEPs) refer to the electrical changes that occur in the peripheral and central portions of the auditory pathway in response to acoustic stimuli (7). Long-latency auditory evoked potentials (LLAEPs) can be subdivided into exogenous potentials, with cortical components termed P1, N1, P2, N2, and P3, and are mainly influenced by the physical characteristics of the stimulus (intensity, frequency, and duration), and endogenous potentials, with the cognitive component P300, which is influenced by internal events related to the cognitive function of the subject (8).

A more thorough evaluation of the hearing of elderly individuals and their difficulties in understanding speech necessarily involves the study of LLAEPs, as they allow the investigation of auditory information processing (attention and memory), that is, the evaluation of the cortical activity involved in acoustic detection, discrimination, and integration skills (9,10). Longer latency responses of the N1 and P2 components of LLAEPs are observed in the elderly population due to temporal changes in the properties of cortical auditory responses, which result in delayed synchronous firing for the perception of acoustic characteristics (11,12).

Recent studies of degenerative diseases have led to the emergence of new concepts, such as mild cognitive impairment (MCI), which typically refers to the clinical stage between normal aging and dementia in its early stage. Changes that occur with MCI may affect the lives of people in various ways. One of the repercussions is the commonly increased difficulty in understanding speech (2).

The Montreal Cognitive Assessment (MoCA) test is a brief cognitive screening tool designed to identify patients with MCI and has been used on a large clinical scale. Approximately 10 min is required to complete this test. It covers eight cognitive domains and has shown very high sensitivity and specificity for MCI (2). The MoCA is a recently developed cognitive test that aims to differentiate between normal aging and MCI and has gained worldwide interest among health professionals due to its high sensitivity (13).

Assessments of late AEPs and analysis of their associations with MoCA results may provide a more in-depth understanding of the aging process and its relationship with cognitive performance and speech comprehension.

Thus, the present study aimed to assess the relationship between cognitive performance and LLAEP in the elderly.

## METHODS

This was a cross-sectional, observational, analytical, prospective study approved by the Ethics Committee of a public university in the state of Alagoas under opinion number 1,774,284.

Two groups were studied: the control group, comprising adults aged 20 to 58 years, and the test group, comprising young elderly individuals between 60 and 70 years of age, according to the World Health Organization (14).

Individuals from both groups were selected according to the following inclusion criterion: auditory threshold equal to or less than 25 dBHL American National Standards Institute (ANSI 1969) (15). Individuals were excluded for the following reasons: otoscopy abnormalities, compromised middle ear, exposure to occupational noise, otologic surgeries, ototoxic medication, tinnitus, vertigo, dizziness or other uncorrected cochleovestibular or visual changes, or motor or neurological disorders severe enough to interfere with the evaluation.

The tests were conducted in a research laboratory at the Public University of the State of Alagoas. Prior to the initiation of the procedures, ethical aspects were addressed by reading the informed consent form, and after acceptance, a collection protocol containing identification data and an auditory history was completed. A hearing screening examination of the participants was carried out beginning with the inspection of the external auditory canal (Welch Allyn^®^ Otoscope Pocket LED), tonal and vocal audiometry (Interacoustics^®^ AC 33 audiometer), and brain stem AEP (Bio-Logic^®^ Navigator Pro). The stimuli were presented in both ears at 80 dBnHL, with a rate of 27.7 stimuli per second. A total of 2,000 stimuli in the rarefied polarity were planned, with a recording window of 12 ms.

After this initial step, the following procedures were performed:

1. For the screening of cognitive functions, the MoCA test, a brief cognitive screening tool, was used to evaluate eight cognitive domains, including visuospatial/executive functions, naming, memory, attention, language, abstraction, delayed recall, and spatiotemporal orientation (16). The highest possible score is 30 points, which represents the best performance. The authors established a cutoff of 26 points, that is, a score of 25 points or below was suggestive of MCI. One point was added to the final score of participants with 12 years or less of formal education to correct for the effect of education level on these individuals (13).
2. To perform the LLAEPs, the volunteers were accommodated in a recliner chair in a supine position in a comfortable manner to allow adequate muscle relaxation. The skin was cleaned with an abrasive paste, and the electrodes were then fixed using a microporous adhesive. An electrolyte paste was used to improve the electrical conductivity. Potential recordings were performed using a Bio-Logic^®^ Navigator Pro device and recorded using five electrodes placed in Fz, Cz (reference), A2 (right) and A1(left), and FPz (ground), using the two recording channels of the equipment. The impedance between the electrodes was kept at a level below 5 kΩ (12,17).

Different stimuli were used to acquire cortical components. Initially, a series of verbal stimuli (syllables /ba/ and /da/) was monoaurally reproduced at an intensity of 80 dBnHL, with a rate of 0.9 stimuli/s and band-pass filters of 1-30 Hz with a gain of 3,000x. Subsequently, nonverbal stimuli (1,000-Hz and 2,000-Hz tones) were presented. Both verbal and nonverbal stimuli were randomly presented at a percentage of 50% each.

The oddball paradigm was used for the acquisition of the cognitive component - the P300 - monoaurally and presented at 20% for rare stimuli (/da/ and 1,000 Hz) and 80% for frequent stimuli (/pa/ and 2,000 Hz).

The examinations were analyzed by two examiners with theoretical and practical knowledge of hearing electrophysiology, especially regarding LLAEPs. To obtain the cortical components, the participants were instructed to listen to sounds in sequence. The participants were also instructed to listen closely and name a rare stimulus (different) whenever it appeared. The tests were analyzed by two evaluators with theoretical and practical knowledge on electrophysiology, especially LLAEPs.

The amplitude and latency values were established according to Melo et al. (18) and Silva et al. (19), which standardized the visualization and marking of waves in the peaks with greater amplitude. In the current study, the latencies and amplitudes of waves P1, N1, P2, N2, and P3, in addition to the cognitive component, were marked following the appearance of the first three waves, initially at the highest peak and then in the negative polarities (positive-negative). The P300 component was located before the highest positive peak, P3, with a latency of approximately 350 ms (20-23).

### Statistical analysis

The data were tabulated and processed using the predictive analytics software microcomputer application PASW^®^ Statistics (IBM SPSS Statistics for MAC, version 23.0, Armonk, NY: IBM Corp.). Tabular and graphical representations of the means and standard deviations were used to describe the data. The normality of the samples was assessed using the Kolmogorov-Smirnov test.

Student’s t-test was used to detect differences between independent numerical variables in the control and experimental groups. In addition, Bonferroni correction was applied to multiple comparisons, and the effect sizes were calculated using Cohen’s d.

Associations between the MoCA test results and the wave, latency, and amplitude components of long-latency potentials were analyzed using the bivariate correlation test, and the degree of linear relationships was determined using Pearson’s coefficient. The alpha values were considered significant at values lower than 0.05. The established beta value was 0.1.

## RESULTS

The sample consisted of 30 participants, 20 adults, and 10 elderly adults. The age of the participants ranged from 20 to 58 years (mean age of 35 years and standard deviation of 13.78 years) for the adult group and between 60 and 70 years (mean age of 64.8 years and standard deviation of 3.45 years) for the elderly adult group. Among all participants, 80% were female and 20% were male.

The Kolmogorov test indicated that the distributions of the groups analyzed were all normal, with *p*-values equal to or greater than 0.354, which enabled the use of parametric tests.

The descriptive results by age group for the MoCA test can be found in Table 1. 

Regarding the MoCA scores, regardless of the age group, participants with normal scores had a mean score of 27.89 points, while those with abnormal scores had a mean score of 24.16 points, with variation rates of 25% in adults and 50% in elderly individuals and a *p*-value of 0.40.

The cortical components, latency and amplitude values, in addition to the association between LLAEP and MoCA scores, are described according to the stimulus used (speech and tone) and age group in Tables 2 and 3.

Thus, when compared by age group, the cortical components (P1, N1, P2, N2, and P3) showed significant differences in the following conditions: (i) signals evoked by the speech stimulus /da/ and the pure-tone stimulus at 2,000 Hz for the N2 amplitude (*p*=0.008 and *p*=0.001, respectively), which were both higher for the adult age group, and (ii) signals evoked by the speech stimulus /da/ for N1 latency (*p*=0.018) and by the pure-tone stimulus at 2,000 Hz for P2 latency (*p*=0.017), with a higher latency for the elderly group.

When comparing the cortical components between adult individuals with normal and abnormal MoCA scores and between elderly individuals with normal and abnormal MoCA scores, no significant differences were found for latencies and amplitudes when signals were evoked with speech or pure-tone stimuli, as indicated by the independent t-test with the necessary Bonferroni corrections.

To correlate cognitive processes and the latency and amplitude of the cortical components, Pearson’s correlation test was applied, which revealed a direct and medium-strength correlation between abnormal MoCA scores and P2 greater amplitude when the speech stimulus /da/ was used (*p*<0.001 and r=0.452).

An analysis of the data per ear was carried out as well as a correlation of the P300 with the cognitive assessment; however, it did not present significant statistical differences (*p*>0.05).

The latency and amplitude values for the cognitive component (P300), are presented in Tables 4 and 5, according to the applied stimulus (tone and speech, respectively), by age group.

## DISCUSSION

### Methods discussion

Considering the importance and usefulness of the neuropsychological tests for cognitive screening that have been translated and validated for the Brazilian population, it was decided to use the Brazilian version of the MoCA, which was validated by Sarmento et al. (24). Another study opted for the Brief Cognitive Screening Battery because it can be rapidly applied and is appropriate for assessing memory in a population with heterogeneous education levels; however, it only evaluates aspects related to memory and attention. In contrast, the MoCA evaluates eight cognitive domains, including visuospatial/executive function, naming, memory, attention, language, abstraction, delayed recall, and spatiotemporal orientation, and is more comprehensive in the evaluation of the language aspects mentioned above (25,26).

In addition, we chose to study the cortical and cognitive components because they represent unique evaluative methods in which electrical potentials are created at various levels of the nervous system in response to acoustic stimulation. However, for the cortical components, a variation of what is commonly applied was utilized, and the different stimuli were presented at a percentage of 50% each. For the cognitive components, the percentage of presentation of rare stimuli was 20%, while that of frequent stimuli was 80% (oddball paradigm), a parameter often used in the literature. The latter is the test that best reflects mental functions and is highly dependent on cognitive abilities, including attention and discrimination (27).

### Results discussion

It is known that for a MoCA, scores between 26 and 30 indicate normal cognition, while scores between 20 and 25 indicate probable MCI, according to national and international studies (28,29). In the study under analysis, 5 adults and 10 elderly people were observed with scores below normal limits.

In the study by Bidelman et al. (30), individuals with possible cognitive impairment exhibited a larger P2 wave amplitude. They suggested that a larger amplitude related to MCI is associated with diffuse changes that occur at different levels of the auditory system, thus interfering with the auditory task and leading to an increase in cortical activation in elderly individuals (31,2). This hypothesis was corroborated by the direct and medium-strength correlation found between abnormal MoCA scores and P2 amplitude in the present study.

Another study (26) reported that the effects of cognitive impairment on cortical and subcortical activities are age-dependent and the increase in the amplitude of potentials, related to both aging and cognitive impairment, suggests diffuse changes in the auditory system.

Lister et al. (32) found that the elderly adults showed a greater amplitude of the P1 component when compared with younger adults; this was attributed to the greater effort required in a sound detection task. In contrast, in the present study, there was no variation in amplitude between the two groups studied. The difference in these findings may be explained by the larger sample size used in the study by Lister et al.

Studies have shown that the cortical component N1 is a marker of the auditory activity of decoding acoustic characteristics and tends to become more prominent and robust throughout development (8,33). Gürkan et al. (34) compared the latencies and amplitudes of the cortical components P1, N1, and P2 in 57 elderly patients with presbycusis and found significant prolongation of the N1 latencies compared with the control group. This finding corroborates the results in the present study, where increased N1 latencies were observed in elderly patients when the speech stimulus was used, reflecting the need for a longer time to process the information provided (35).

Another important factor is that elderly individuals usually present lower cortical responses to pure-tone stimuli than younger adults (36). The reduced N2 amplitude in the elderly population is due to cognitive impairment in these individuals, necessitating a greater attentional effort to discriminate the stimulus presented, which corroborates the results found in this study.

Regarding the increased latency of the P2 component in the elderly group compared to adults without cognitive impairment, studies indicate that this component is influenced by the level of attention of the participant to the stimulus presented, which proves the need for more time for the physiological process of sound discrimination in this group (37,38).

The cognitive component P300 presented a higher latency in the elderly group in the present study (Table 5). This is similar to another study by Krishnamurti et al. (39) to assess the latencies of the P300 component in adults and elderly individuals with auditory processing disorders which found increased P300 latencies in the elderly group. The increase in latency reflects a slow process of auditory information searching and processing (40) and the possibility of monitoring speech therapy because this parameter is relatively quick and inexpensive to evaluate compared to behavioral tests for assessing central auditory processing (41).

Thus, the main analyses for the elderly group described in the present study refer to the decrease in N2 amplitude, both with speech stimulus and a pure-tone of 2,000 Hz; increased N1 latency with speech stimulus; increased P2 latency with the pure-tone stimulus at 2,000 Hz; and increased P300 latency with speech stimulus. Finally, there was a direct and medium-strength correlation between abnormal scores obtained on the MoCA and P2 amplitude, which suggests diffuse changes in the auditory system and increased cortical activation (26,30).

The theme addressed represents an important contribution to the audiological diagnosis of age-related communicative disorders. The results of the study provide a better understanding of the physiological processes involved and favor a more detailed investigation of the auditory and linguistic functions of the elderly population.

## CONCLUSION

There is a relationship between the long-latency potentials and cognitive performance in the elderly population, which was observed by an increased P2 amplitude and by impairment of the sound decoding process.

## AUTHOR CONTRIBUTIONS

Oliveira MFF was the principal researcher, responsible for the research elaboration, literature survey, data collection and analysis. Menezes PL was the advisor, responsible for the data analysis, manuscript writing and correction. Carnaúba ATL was responsible for manuscript writing, preparation of the schedule, submission, and procedures of the archives. Pereira LD was responsible for the manuscript grammar correction. Andrade KCL was responsible for data collection and analysis. Frizzo ACF was responsible for the data analysis and approval of the final version of the manuscript. Soares IA was the advisor, responsible for the research elaboration, data analysis, and approval of the final version of the manuscript.

## Figures and Tables

**Table 1:** Descriptive statistics of the MoCA scores (in points).

	Group	Mean	SD	Higher CI	Lower CI	Cohen’s d	*p*-value/t
	Adult	25.89	2.48	26.71	25.08		
MoCA						0.04	0.65/2.39
	Elderly adult	24.22	2.77	25.60	22.84		

Note: Analysis of the independent t-test

Abbreviations: MoCA, Montreal Cognitive Assessment; CI, 95% confidence interval; SD, standard deviation

**Table 2:** Values of latency (ms) and amplitude (µV) of the cortical components with speech stimuli by age group.

Cortical component	Adults		Elderly adults				
	Mean	SD	Mean	SD	Cohen’s d	T value	*p*-valueAge group
Latency - /pa/							
P1	69.94	13.97	74.66	11.30	0.375	-0.634	0.510
N1	122.27	18.36	126.66	14.79	0.265	-0.194	0.856
P2	209.03	40.80	234.81	37.24	0.641	-1.362	0.201
N2	276.37	31,01	289.17	52.72	0.296	0.717	0.326
P3	335.60	58.81	351.86	56.51	0.280	-0.784	0.431
Latency - /da/							
P1	64.49	23.70	65.58	17.96	0.052	-0.713	0.479
N1	113.20	25.13	130.88	33.08	0.081	-2.430	0.018
P2	208.48	45.87	227.29	58.22	0.421	-1.015	0.318
N2	262.28	61.80	269.38	61.81	0.114	-0.196	0.845
P3	339.78	64.92	330.81	57.66	0.145	0.377	0.708
Amplitude - /pa/							
P1	1.90	2.39	2.49	1.22	0.316	0.749	0.457
N1	-4.93	2.31	-6.11	2.64	0.480	1.173	0.249
P2	2.73	1.71	2.84	2.10	0.036	0.541	0.592
N2	1.55	2.75	-0.57	1.02	1.021	-1.239	0.220
P3	2.29	1.48	2.36	1.16	0.052	1.285	0.205
Amplitude - /da/							
P1	1.90	1.57	1.63	1.78	0.160	-0.905	0.372
N1	-3.75	2.04	-4.83	2.03	0.540	1.392	0.172
P2	2.54	1.94	2.23	1.98	0.163	0.957	0.345
N2	-1.99	2.36	0.26	1.68	1.098	-2.771	0.008*
P3	2.19	1.71	1.54	1.40	0.410	0.030	0.976

Note: Analysis of the independent t-test

Abbreviation: SD, standard deviation

* Statistically significant

**Table 3:** Values of the latency (ms) and amplitude (µV) of the cortical components with tone stimuli at 1,000 Hz and 2,000 Hz by age group.

Cortical component	Adults		Elderly adults				
	Mean	SD	Mean	SD	Cohen’s d	T value	*p*-valueAge group
Latency - 1,000 Hz							
P1	57.88	11,89	57.89	1117	0.009	0.973	0.337
N1	107.72	27.00	108.55	13.54	0.037	-0.515	0.609
P2	188.67	39.63	202.53	37.56	0.363	-1.415	0.164
N2	254.64	57.00	272.86	48.03	0.516	-1.145	0.259
P3	326.25	59.93	322.27	50.98	0.072	0.184	0.854
Latency - 2,000 Hz							
P1	57.01	13.51	57.08	9.02	0.008	0.009	0.993
N1	106.16	17.03	102.11	14.92	0.250	0.901	0.372
P2	188.97	29.76	209.71	39.14	0.605	-2.532	0.017*
N2	260.94	48.13	269.04	44.44	0.174	-0.639	0.527
P3	322.28	53.90	326.13	62.00	0.067	-0.166	0.870
Amplitude - 1,000 Hz							
P1	0.80	1.56	0.75	0.99	0.080	0.722	0.473
N1	-4.08	2.66	-3.70	2.28	0.124	-1.036	0.307
P2	3.15	2.23	3.01	2.08	0.071	-1.145	0.465
N2	-1.48	1.87	-0.98	2.17	0.296	-0.656	0.516
P3	1.87	1.81	1.06	1.45	0.532	1.892	0.065
Amplitude - 2,000 Hz							
P1	1.01	1.22	1.46	0.91	0.433	-1.204	0.235
N1	-3.81	2.32	-4.13	2.46	0.127	0.481	0.663
P2	2.97	1.93	3.15	2.07	0.102	0.205	0.839
N2	-1.41	1.64	-0.29	0.90	0.846	-3.373	0.001*
P3	1.52	1.64	1.41	0.92	0.083	0.129	0.898

Note: Analysis of the independent t-test

Abbreviation: SD, standard deviation

* Statistically significant

**Table 4:** Latency (ms) and amplitude (µV) values of the P300 component, with tone stimulus, by age group.

Cognitive component	Group	Mean	SD	Higher CI	Lower CI	*p*-value
	Adult	361.97	39.45	374.94	349.00	
P300 (Latency)						0.510
	Elderly adult	362.99	55.37	390.52	335.45	
	Adult	5.59	3.58	6.77	4.41	
P300 (Amplitude)						0.770
	Elderly adult	4.22	2.08	5.26	3.18	
						

Note: Analysis of the independent t-test

Abbreviations: ms, milliseconds; µV, microvolts; CI, confidence interval; SD, standard deviation

**Table 5:** Latency (ms) and amplitude (µV) values of the P300 component, with speech stimulus, by age group.

Cognitive component (P300) - speech	Group	Mean	SD	Higher CI	Lower CI	*p*-value
	Adults	355.92	41.49	369.56	342.28	
Latency (ms)						0.013*
	Elderly adults	381.10	35.22	398.61	363.58	
	Adults	5.08	3.02	6.07	4.09	
Amplitude (μV)						0.318
	Elderly adults	6.12	3.21	7.72	4.52	

Note: Analysis of the independent t-test

Abbreviations: ms, milliseconds; µV, microvolts; CI, confidence interval; SD, standard deviation

* significant p and Cohen’s d=0.660
